# Supplementary material for: Demonstrating paths for unlocking the value of cloud genomics through cross cohort analysis
Source: Nat Commun. 2023 Sep 5;14:5419. doi: 10.1038/s41467-023-41185-x (PMC10480504; doi:10.1038/s41467-023-41185-x)
Supplement: Supplementary file 3 — Description of Additional Supplementary Files [file 41467_2023_41185_MOESM3_ESM.pdf]

Description of Additional Supplementary Files

File Name: Supplementary Data 1
Description: Lead variants Meta-analysis: Summary statistics reported were obtained from two-sided genetic association testing performed using REGENIE and meta-analyzed using METAL.

File Name: Supplementary Data 2
Description: Lead variants Pooled: Summary statistics reported were obtained from two-sided genetic association testing performed using REGENIE.

File Name: Supplementary Data 3
Description: Number of variants in each ancestry (Percentage)
