# Supplementary material for: Demonstrating paths for unlocking the value of cloud genomics through cross cohort analysis
Source: Nat Commun. 2023 Sep 5;14:5419. doi: 10.1038/s41467-023-41185-x (PMC10480504; doi:10.1038/s41467-023-41185-x)
Supplement: Supplementary file 5 — Reporting Summary [file 41467_2023_41185_MOESM5_ESM.pdf]

nature portfolio

Corresponding author(s): Alexander G. Bick

Last updated by author(s): Jul 15, 2023

Reporting Summary

Nature Portfolio wishes to improve the reproducibility of the work that we publish. This form provides structure for consistency and transparency in reporting. For further information on Nature Portfolio policies, see our Editorial Policies and the Editorial Policy Checklist.

Statistics

For all statistical analyses, confirm that the following items are present in the figure legend, table legend, main text, or Methods section.

n/a	Confirmed	
☐	☒	The exact sample size (*n*) for each experimental group/condition, given as a discrete number and unit of measurement
☐	☒	A statement on whether measurements were taken from distinct samples or whether the same sample was measured repeatedly
☐	☒	The statistical test(s) used AND whether they are one- or two-sided<br>*Only common tests should be described solely by name; describe more complex techniques in the Methods section.*
☐	☒	A description of all covariates tested
☐	☒	A description of any assumptions or corrections, such as tests of normality and adjustment for multiple comparisons
☐	☒	A full description of the statistical parameters including central tendency (e.g. means) or other basic estimates (e.g. regression coefficient) AND variation (e.g. standard deviation) or associated estimates of uncertainty (e.g. confidence intervals)
☐	☒	For null hypothesis testing, the test statistic (e.g. *F*, *t*, *r*) with confidence intervals, effect sizes, degrees of freedom and *P* value noted<br>*Give P values as exact values whenever suitable.*
☒	☐	For Bayesian analysis, information on the choice of priors and Markov chain Monte Carlo settings
☒	☐	For hierarchical and complex designs, identification of the appropriate level for tests and full reporting of outcomes
☐	☒	Estimates of effect sizes (e.g. Cohen's *d*, Pearson's *r*), indicating how they were calculated

Our web collection on statistics for biologists contains articles on many of the points above.

Software and code

Policy information about availability of computer code

Data collection	UK Biobank (UKB) 200K Exomes, All of Us 98K WGS data, gnomAD v3.1.2.
Data analysis	The code for all analyses can be found in https://github.com/all-of-us/ukb-cross-analysis-demo-project and was compatible with UK Biobank Research Analysis Platform and All of Us Researcher Workbench available data and technical capabilities as of the Spring of 2022. Other software: PLINK(2.3.Alpha), Hail version 0.2.74-0c3a74d12093, REGENIE(v2.2.4), METAL(version released on 2011-03-25), Python(version 3.x), R(versions 3.x and 4.x)

For manuscripts utilizing custom algorithms or software that are central to the research but not yet described in published literature, software must be made available to editors and reviewers. We strongly encourage code deposition in a community repository (e.g. GitHub). See the Nature Portfolio guidelines for submitting code & software for further information.

Data

Policy information about availability of data

All manuscripts must include a data availability statement. This statement should provide the following information, where applicable:

- Accession codes, unique identifiers, or web links for publicly available datasets
- A description of any restrictions on data availability
- For clinical datasets or third party data, please ensure that the statement adheres to our policy

The UK Biobank (UKB) whole-exome sequence data can be accessed through UKB Research Analysis Platform (RAP), through the UKB approval system (https://

www.ukbiobank.ac.uk). Access to individual-level data from the All of Us research program is available to researchers whose institution has signed a data use agreement with All of Us (https://www.researchallofus.org/register/). Whole-genome sequencing data belongs to the controlled tier dataset, which requires additional training to access. gnomAD is publicly available (https://gnomad.broadinstitute.org/).

Research involving human participants, their data, or biological material

Policy information about studies with human participants or human data. See also policy information about sex, gender (identity/presentation), and sexual orientation and race, ethnicity and racism.

Reporting on sex and gender	We did not carry out sex-specific analysis. Sex was a covariate in our analysis.
Reporting on race, ethnicity, or other socially relevant groupings	Self reported race was used as covariate in the models.
Population characteristics	Multi-ancestral cohort from All of Us and UKB were used in the analysis. Age, Sex and Race were used as additional covariates
Recruitment	Each cohort recruited participants, details are provided in the supplementary text.
Ethics oversight	UKB: UK Biobank has approval from the North West Multi-centre Research Ethics Committee (MREC).<br>AOU: Institutional Review Board (IRB) of the All of Us Research Program. The All of Us IRB follows the regulations and guidance of the NIH Office for Human Research Protections.<br>Massachusetts General Hospital, IRB.

Note that full information on the approval of the study protocol must also be provided in the manuscript.

Field-specific reporting

Please select the one below that is the best fit for your research. If you are not sure, read the appropriate sections before making your selection.

☒ Life sciences ☐ Behavioural & social sciences ☐ Ecological, evolutionary & environmental sciences

For a reference copy of the document with all sections, see nature.com/documents/nr-reporting-summary-flat.pdf

Life sciences study design

All studies must disclose on these points even when the disclosure is negative.

Sample size	This study is conducted primarily to investigate plasma lipids from whole genome sequenced All of Us and whole exome 200K sequenced dataset. We included all the samples with plasma lipid measure (All of Us: 37,754 and UKB: 190982). We did not carry out independent sample-size calculations in this study and choose to include all samples to maximize the cohort size.
Data exclusions	All samples and variants passing the central QC were included in this study.
Replication	This is a workflow/pipeleine development project, therefore replication was not carried out.
Randomization	No randomization was performed. Since this is a population based study and did not focus on a treatment effect, randomization was not performed.
Blinding	No blinding was performed. Blinding was not performed as there was no randomization. Investigators did not have any access to identifying information.

Reporting for specific materials, systems and methods

We require information from authors about some types of materials, experimental systems and methods used in many studies. Here, indicate whether each material, system or method listed is relevant to your study. If you are not sure if a list item applies to your research, read the appropriate section before selecting a response.

Materials & experimental systems

n/a	Involved in the study
☒	☐ Antibodies
☒	☐ Eukaryotic cell lines
☒	☐ Palaeontology and archaeology
☒	☐ Animals and other organisms
☒	☐ Clinical data
☒	☐ Dual use research of concern
☒	☐ Plants

Methods

n/a	Involved in the study
☒	☐ ChIP-seq
☒	☐ Flow cytometry
☒	☐ MRI-based neuroimaging
